# Paroxysmal, Persistent, and Permanent Type-1 Brugada Pattern: Does Burden Matter?

**DOI:** 10.3390/jcdd12020065

**Published:** 2025-02-10

**Authors:** Thanaboon Yinadsawaphan, Pattara Rattanawong, Narathorn Kulthamrongsri, Win-Kuang Shen, Dan Sorajja

**Affiliations:** 1 Department of Cardiovascular Medicine, Mayo Clinic, Phoenix, AZ 85054, USA; thanaboon.yin@gmail.com (T.Y.); kulthamrongsri.narathorn@mayo.edu (N.K.);; 2 Department of Medicine, John A. Burns School of Medicine, University of Hawai’i, Honolulu, HI 96813, USA; 3 Pali Momi Heart Center, Hawaii Pacific Health, Honolulu, HI 96701, USA

**Keywords:** Brugada, spontaneous type-1 Brugada, major arrhythmic event, arrhythmia, burden

## Abstract

Spontaneous type-1 Brugada patterns are associated with an elevated risk of major arrhythmic events, yet the relationship between varying degrees of pattern burden and the occurrence of a first major arrhythmic event remains unclear. This retrospective cohort study included 64 adult patients with a spontaneous type-1 Brugada pattern, who were identified at Mayo Clinic sites and followed for ≥12 months after the initial diagnosis. All patients underwent at least three 12-lead electrocardiograms (ECGs) within the first year. Individuals with prior major arrhythmic events were excluded. The percentage of ECGs showing a type-1 pattern was calculated and categorized as paroxysmal (<50%), persistent (50–99%), or permanent (100%). During a median follow-up of 92 months, seven patients (11%) experienced their first major arrhythmic event. Of these, one had paroxysmal, four had persistent, and two had permanent spontaneous type-1 Brugada patterns. Although statistical significance was not reached, the hazard ratios suggested a trend toward increased risk with persistent and permanent patterns compared to paroxysmal patterns. No sudden cardiac deaths occurred during follow-up. These findings suggest that a higher burden of spontaneous type-1 Brugada patterns may be associated with increased arrhythmic risk.

## 1. Introduction

Risk stratification in Brugada Syndrome remains challenging due to the variability in arrhythmic risk across patient populations. Current efforts focus on identifying patients at high risk of sudden cardiac death or major arrhythmic events while avoiding overtreatment in low-risk individuals. The spontaneous type-1 Brugada pattern is a well-known risk factor associated with major arrhythmic events in Brugada syndrome [1]. A recent study reported fluctuations in patients with spontaneous type-1 Brugada patterns during follow-up and evaluated the association between the burden of the type-1 Brugada pattern on 12-lead Holter monitoring and the history of major arrhythmic events [2]. However, no studies have explored the specific association between the burden of spontaneous type-1 Brugada pattern and the occurrence of first major arrhythmic events. Therefore, our study aims to define the burden of type-1 Brugada patterns and assess their association with patients’ first major arrhythmic events.

## 2. Methods

### 2.1. Study Design and Population

This retrospective cohort study included 64 patients with spontaneous type-1 Brugada pattern. All study protocols were approved by the Institutional Review Board of Mayo Clinic.

The population in this study was a part of the previously published Brugada registry at the Mayo Clinic [3]. In brief, this study attempted to include all adult patients (age ≥ 18 years) with a spontaneous type-1 Brugada pattern encountered at 3 hospitals and 15 community and specialty clinic sites at Mayo Clinic Campuses (Arizona: Phoenix; Scottsdale, Florida: Jacksonville; and Minnesota: Rochester) and 19 hospitals and 65 community and specialty clinic sites at Mayo Clinic Health System (Minnesota: 29 cities; Iowa: 2 cities; and Wisconsin: 18 cities).

In the original Brugada registry, the enrollment protocol started with the identification of all patients with any of the following documented keywords: ‘Brugada pattern, Brugada electrocardiogram, Brugada syndrome, type-1 Brugada pattern, or Brugada Type-1’, found in Mayo Clinic medical records, including electrocardiogram (ECG) reading results, between 1 January 1992, and 31 December 2021. We subsequently retrieved all ECGs of those patients and performed a manual review to identify patients who had at least one ECG with a type-1 Brugada pattern, according to the standard definition [4], performed at Mayo Clinic. Known Brugada syndrome patients who were referred from out-of-network hospitals without an ECG performed at Mayo Clinic were excluded from the study.

The inclusion criteria of this study were as follows: (1) adults (age ≥18 years) with spontaneous type-1 Brugada pattern, (2) followed up at Mayo Clinic for ≥12 months, and (3) had ≥three 12-lead electrocardiograms documented in the first 12 months after the first spontaneous type-1 Brugada pattern diagnosis. To clarify, this study did not include patients with a type-1 Brugada pattern associated with other etiologies such as fever, exercise, electrolyte abnormalities, conductive diseases (Lyme disease, Long QT syndrome), chest contusion, and known substances and medications leading to the type-1 Brugada pattern. We excluded all patients who had experienced any major arrhythmic events prior to the first documented type-1 Brugada ECG at Mayo Clinic.

The demographic information was obtained by manually reviewing patients’ charts at time zero, which was defined as the date of the first documented ECG with the spontaneous type-1 Brugada pattern in the Mayo Clinic Network. This study followed the patients from the first documented ECG with spontaneous type-1 Brugada pattern until January 2023.

### 2.2. Brugada Burden

All ECGs performed at Mayo Clinic after the date of the first ECG with a type-1 Brugada pattern until the last follow-up date of each patient were retrieved and reviewed manually. The definition of the type-1 Brugada pattern was strictly followed in accordance with Heart Rhythm Society guidelines [4] and consensus statement diagnostic criteria [5]. The burden of type-1 Brugada pattern was calculated as shown below Equation (1).

(1) $$Brugada\;burden\;(\%)=\frac{the\;number\;of\;ECGs\;with\;type\;1\;Brugada\;pattern}{the\;total\;number\;of\;ECGs\;after\;the\;first\;type\;1\;Brugada\;pattern\;}$$

The patients were divided retrospectively into three groups based on Brugada burden: paroxysmal (<50%), persistent (≥50%), and permanent (100%).

### 2.3. Outcome

The main outcome of this study was the first major arrhythmic event after diagnosis of the spontaneous type-1 Brugada pattern. Major arrhythmic events were defined as sustained ventricular arrhythmias, appropriate defibrillator therapy, sudden cardiac arrest, or sudden cardiac death [6].

### 2.4. Statistical Analysis

All continuous variables were tested for normality and were reported as a mean ± SD or median (Q1–Q3). Differences in continuous variables between 3 groups were tested using one-way analysis of variance (ANOVA) or a Kruskal–Wallis test as appropriate. Differences in categorical variables were tested using Pearson’s chi-square. Significant variables subsequently underwent pairwise post hoc testing with adjusted *p* values via Bonferroni correction for multiple tests.

The incidence rate per person-year was calculated by dividing the number of arrhythmic events by the total person-years of observation in each group. Poisson regression was used to compute the incidence rate ratio.

One minus the Kaplan–Meier survival function was selected to compute the cumulative probability of a patient experiencing their first arrhythmic event over the follow-up time. A Cox proportional hazards model was used to compare the time to first arrhythmic event between patients with a paroxysmal, persistent, and permanent type-1 Brugada pattern. We subsequently compared the cumulative probability of the first arrhythmic event in patients with a low Brugada burden (<50%) and a high Brugada burden (≥50%).

All statistical tests were performed by IBM SPSS Statistics version 29.0.2.0 (IBM Corporation, Armonk, NY, USA). *p*-values of <0.050 were considered statistically significant for all analyses.

## 3. Results

### 3.1. Patient Population and Baseline Characteristics

Between January 1992 and December 2021, a total of 110 patients with spontaneous type-1 Brugada pattern were identified at three Mayo Clinic sites (Arizona, Florida, and Rochester). Of these, 64 patients met the inclusion criteria of having at least 12 months of follow-up and a minimum of three separate 12-lead ECG recordings in the first year after diagnosis. The included patients were predominantly male (78%), with a mean age of 49.5 ± 14.7 years; the majority were White (88%), followed by Asian (6%) and Black (5%) patients (Table 1).

At baseline, the distribution of type-1 Brugada pattern burden, defined by the percentage of ECGs demonstrating a spontaneous type-1 pattern over the follow-up period, was as follows: paroxysmal (<50% burden) in 19 patients (30%), persistent (50–99% burden) in 20 patients (31%), and permanent (100% burden) in 25 patients (39%).

Eight patients (12.5%) had a history of cardiac syncope and nine patients (14%) had a family history of sudden cardiac death. Eighteen patients underwent genetic testing, of whom ten (56%) tested positive for an SCN5A mutation. An implantable cardioverter-defibrillator (ICD) was placed in 16 patients (25%) for primary prevention.

### 3.2. Follow-Up and Major Arrhythmic Events

The median follow-up was 92 months (Q1–Q3 33–153 months). Seven patients (11%) experienced their first major arrhythmic event during follow-up, which included three sustained ventricular arrhythmias, three appropriate ICD therapies, and one sudden cardiac arrest. Among these seven patients, only one patient exhibited a paroxysmal type-1 Brugada pattern with a type-1 burden of 17%, four patients had persistent patterns (type-1 burdens ranging from 50% to 83%), and two patients had a permanent pattern. Two of the seven patients experienced two major arrhythmic events during follow-up. The type of major arrhythmic events was demonstrated in Table 2. No death occurred during follow-up.

The crude incidence rates of major arrhythmic events were 0.4%, 2.6%, and 2.1% per person-year in patients with paroxysmal, persistent, and permanent type-1 Brugada patterns, respectively. When compared to patients with paroxysmal type-1 Brugada pattern, the incidence rate ratios of major arrhythmic events in patients with persistent, and permanent type-1 Brugada patterns were 4.9 (95% CI: 0.6–43.0; *p* = 0.45) and 2.5 (95% CI: 0.2–26.7; *p* = 0.15), respectively.

### 3.3. Risk Comparison of First Major Arrhythmic Event

**Figure 1:**
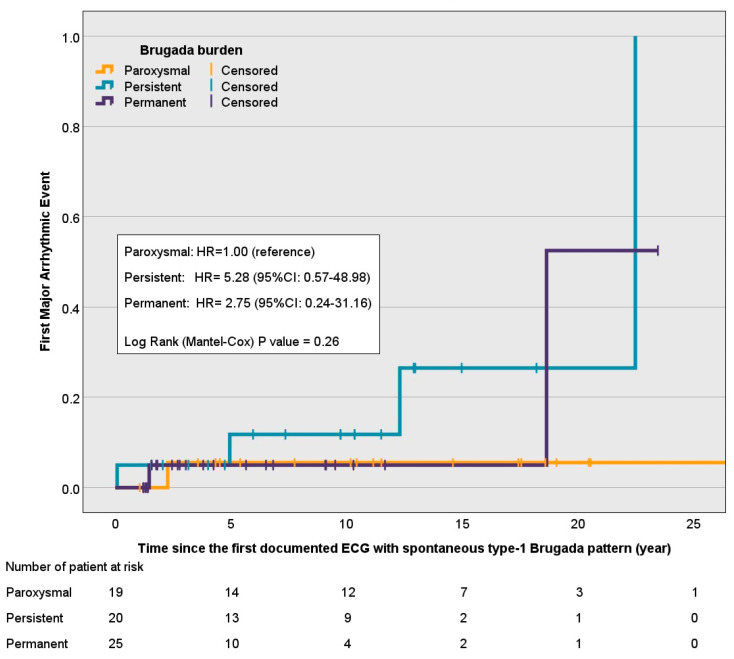
One minus survival functions of time to first major arrhythmic event stratified by Brugada burden categories: paroxysmal (<50%), persistent (≥50% but <100%), and permanent (100%).

**Figure 2:**
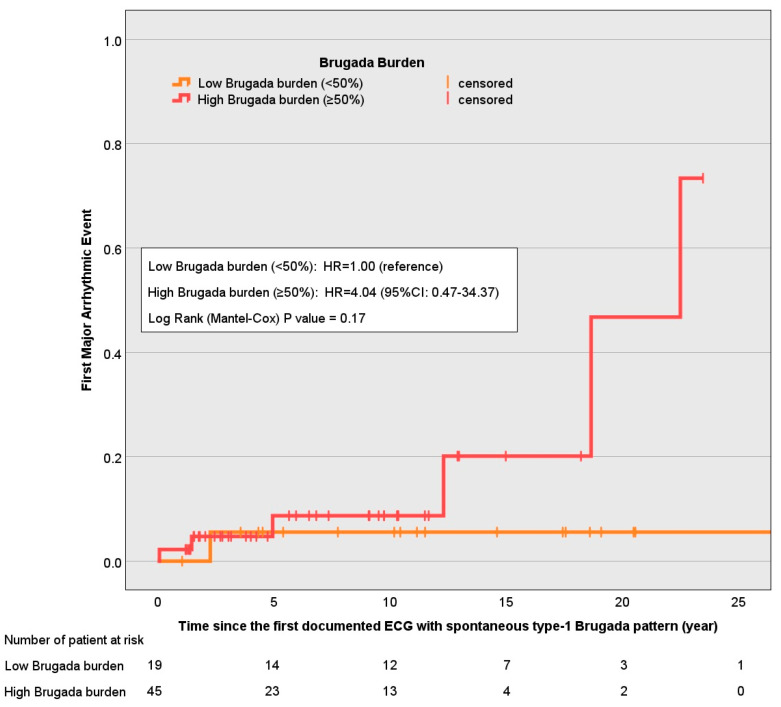
One minus survival functions of time to first major arrhythmic event by high (≥50%) and low (<50%) Brugada burden.

Survival analyses showed a non-significant increase in risk (*p*-value = 0.26) of a first major arrhythmic event among patients with persistent/permanent spontaneous Brugada Type-1 patterns compared to the paroxysmal pattern (Figure 1). In a Cox proportional hazards model, the hazard ratio (HR) for persistent patterns was 5.28 (95% CI: 0.57–48.98), and for permanent patterns it was 2.75 (95% CI: 0.24–31.16), using the paroxysmal pattern as a reference. Similarly, when dichotomized into low (<50%) versus high (≥50%) type-1 burden, there was a trend toward increased risk in the high-burden group (HR = 4.04; 95% CI: 0.47–34.37), although this did not achieve statistical significance (*p*-value = 0.17), Figure 2.

## 4. Discussion

Spontaneous type-1 Brugada ECG pattern was described as a significant predictor of major arrhythmic events, with studies demonstrating its association with a two-fold increased risk compared to drug-induced Brugada patterns [7,8,9]. The consistent finding holds for both asymptomatic and symptomatic Brugada individuals, and it remains a significant predictor of arrhythmic events in multivariate analysis [1]. However, not every patient consistently shows a spontaneous type-1 Brugada pattern when their ECGs are repeatedly analyzed [10].

It is interesting that only about one-third of our patients showed a permanent type-1 Brugada pattern, one-third showed a persistent type-1 Brugada pattern, and one-third showed a paroxysmal type-1 Brugada pattern. A recent meta-analysis of 19 studies involving 5112 patients showed an inconsistent degree of association between spontaneous type-1 Brugada pattern and the risk of major arrhythmic events. The odds ratio ranged from 0.43 to 39.86, with a pooled odds ratio of 1.71 and significant heterogeneity between the included studies [7]. We propose that the considerable heterogeneity between studies is likely due to the different degrees of the burden of the type-1 Brugada pattern.

The concept of Brugada burden and its impact on clinical outcomes has been minimally explored, despite the established dynamic nature of the type-1 Brugada ECG pattern. The spontaneous type-1 Brugada pattern demonstrates significant daily and circadian fluctuations, with an increased prevalence in the evening and after meals [11,12]. Previous studies showed that some patients initially diagnosed with a drug-induced type-1 Brugada pattern revealed a spontaneous type-1 Brugada pattern during 12-lead Holter monitoring or repeated ECGs [2,13]. Similarly, Gray et al. reported that patients with arrhythmic events exhibited a longer burden of ST-segment elevation during 24 h Holter monitoring compared to event-free patients [2]. This finding suggests that quantifying Brugada burden could impact risk stratification.

The incidence rate of major arrhythmic events in our study is similar to findings from a large prospective study of asymptomatic patients with Brugada ECG patterns. In that study, the annual incidence of arrhythmic events for individuals with spontaneous type-1 Brugada ECG was reported to be 0.4% [14]. Similarly, our findings demonstrated that patients with paroxysmal spontaneous type-1 Brugada patterns had an incident rate matching this value at 0.4%. However, patients with persistent and permanent patterns in our cohort exhibited higher incidence rates of arrhythmic events at 2.6% and 2.1% per person-year, respectively, and higher hazard ratios of first major arrhythmic event development, although the differences were not statistically significant. Notably, one patient with a paroxysmal type-1 Brugada pattern experienced sudden cardiac arrest. While a lower Brugada burden tends to be associated with a lower incidence of major arrhythmic events, it does not entirely eliminate the risk of life-threatening arrhythmias.

Current guidelines recommend ICD implantation as a form of secondary prevention for spontaneous type-1 Brugada patients with a history of major arrhythmic events. However, the decision to implement an ICD placement for primary prevention in asymptomatic patients remains challenging [15]. The potential risk of sudden cardiac death must be carefully weighed against the risk of device-related complications. Our study suggests that a higher burden of spontaneous type-1 Brugada patterns may be associated with an increased risk of first major arrhythmic events. If confirmed in future studies, these findings could enhance risk stratification and guide ICD implantation decisions, helping to identify asymptomatic patients with spontaneous type-1 Brugada ECG who are most likely to benefit while minimizing unnecessary interventions.

This study has several limitations. First, although the population in this study is larger than in previously mentioned studies [2,13], the sample size is not large enough and lacks the statistical power to detect significant differences between groups. Based on sample size calculations for cohort studies obtained using Statcalc [16], with an estimated 5.3% outcome rate in the unexposed group and 13.3% in the exposed group, a total of 525 patients with a spontaneous type-1 Brugada pattern would be required to achieve statistical significance. Due to the rarity of Brugada syndrome, a multicenter approach is necessary to enroll a sufficient sample size and achieve the statistical power needed to validate the association between Brugada pattern burden and major arrhythmic events. Second, the burden of the type-1 Brugada pattern in this study was calculated using 12-lead ECGs after diagnosis. While this provides some insight, it is less precise than 12-lead Holter monitoring, which can capture the dynamic and temporal fluctuations of the Brugada pattern more effectively. Additionally, repeated ECGs in this study did not account for the timing of recording, even though prior studies have shown that evening ECGs are more likely to detect a type-1 Brugada pattern due to increased vagal tone in the evening. Another limitation is the predominantly White patient population, which limits the generalizability of our findings to other ethnic groups. Finally, the shorter follow-up duration in the permanent type-1 Brugada pattern group restricted our ability to detect the first arrhythmic events. Despite these limitations, this study serves as a pilot study for the Brugada burden and its impact on major arrhythmic outcome.

## 5. Conclusions

Our study showed possible association trends between the burden of 50% or more type-1 Brugada patterns and the first major arrhythmic events. A larger study with 12-lead Holter monitoring with a long-term follow-up is needed to confirm this observation. This study could lead to a new definition of spontaneous type-1, as it informs the classification of paroxysmal (<50%), persistent (≥50%), and permanent (100%) type-1 Brugada patterns to facilitate a better prognostic evaluation.

## Figures and Tables

**Table 1:** Baseline characteristics of the patients stratified by spontaneous type-1 Brugada burden.

Parameter	Total (*n* = 64)	Paroxysmal (*n* = 19)	Persistent (*n* = 20)	Permanent (*n* = 25)	*p*-Value
Male, n (%)	50 (78.1%)	14 (73.7%)	18 (90.0%)	18 (72.0%)	0.30
Age (years), mean ± SD	49.5 ± 14.7	51.7 ± 15.4	50 ± 16.4	46.7 ± 12.8	0.50
Race, n (%)					0.69
White	56 (87.5%)	17 (89.5%)	18 (90.0%)	21 (84.0%)	
Asian	4 (6.3%)	2 (10.5%)	1 (5.0%)	1 (4.0%)	
African American	3 (4.7%)	0 (0.0%)	1 (5.0%)	2 (8.0%)	
Unknown	1 (1.6%)	0 (0.0%)	0 (0.0%)	1 (4.0%)	
Ethnicity, n (%)					0.532
Hispanic or Latino	2 (3.1%)	1 (5.3%)	1 (5.0%)	0 (0.0%)	
Not Hispanic or Latino	58 (90.6%)	18 (94.7%)	17 (85.0%)	23 (92.0%)	
Unknown	4 (6.3%)	0 (0.0%)	2 (10.0%)	2 (8.0%)	
BMI, mean ± SD	27.9 ± 6.8	29.7 ± 9.0	24.6 ± 3.8	31.1 ± 3.6	0.10
LVEF (%), mean ± SD	62.9 ± 5.4	63.4 ± 6.9	63.6 ± 4.5	61.6 ± 4.6	0.52
Systolic blood pressure (mmHg), mean ± SD	123 ± 17	128 ± 12	123 ± 16	120 ± 19.9	0.26
Diastolic blood pressure (mmHg), mean ± SD	74 ± 9	76 ± 8	71 ± 10	74 ± 10	0.34
Heart rate (beats per minute), mean ± SD	74 ± 18	72 ± 10	70 ± 15	79 ± 18	0.12
Diabetes, n (%)	9 (14.1%)	3 (15.8%)	4 (20.0%)	2 (8.0%)	0.50
Hypertension, n (%)	18 (28.1%)	7 (36.8%)	6 (30.0%)	5 (20.0%)	0.46
Dyslipidemia, n (%)	15 (23.4%)	6 (31.6%)	4 (20.0%)	5 (20.0%)	0.61
Chronic kidney disease, n (%)	2 (3.1%)	1 (5.3%)	1 (5.0%)	0 (0.0%)	0.52
Ischemic cardiomyopathy, n (%)	1 (1.6%)	1 (5.3%)	0 (0.0%)	0 (0.0%)	0.30
Non-ischemic cardiomyopathy, n (%)	0 (0.0%)	0 (0.0%)	0 (0.0%)	0 (0.0%)	n/a
Family history of sudden cardiac arrest, n (%)	9 (14.1%)	3 (15.8%)	4 (20.0%)	2 (8%)	0.50
Family history of Brugada, n (%)	4 (6.3%)	1 (5.3%)	2 (10.0%)	1 (4.0%)	0.70
SCN5A mutation, n (%)	10/18 (55.6%)	3/4 (75.0%)	4/6 (66.7%)	3/8 (37.5%)	0.37
Syncope, n (%)					0.49
Cardiac syncope	8 (12.5%)	3 (15.8%)	4 (20.0%)	1 (4.0%)	
Vasovagal syncope	6 (9.4%)	1 (5.3%)	1 (5.0%)	4 (16.0%)	
Unclear etiology	2 (3.1%)	1 (5.3%)	0 (0.0%)	1 (4.0%)	

**Table 2:** Outcomes and incidences of major arrhythmic events by spontaneous type-1 Brugada burden.

**Parameter**	Total (*n* = 64)	Paroxysmal (*n* = 19)	Persistent (*n* = 20)	Permanent (*n* = 25)	*p* Value
Follow-up time (months), median (Q1–Q3)	92 (33–153)	135 (55–226)	121 (51–158)	37 (19–111)	<0.01 (1.00 ^a^, <0.01 ^b^, 0.04 ^c^) *
Number of ECG, median (Q1–Q3)	5 (3–7)	6 (4–10)	6 (3–8)	4 (3–5)	0.03 (0.73 ^a^, 0.02 ^b^, 0.43 ^c^) *
ICD implantation	16 (25.0%)	5 (26.3%)	8 (40.0%)	3 (12.0%)	0.10
Major arrhythmic events	7 (10.9%)	1 (5.3%)	4 (20.0%)	2 (8.0%)	0.28
Number of major arrhythmic events					0.54
1	5 (7.8%)	1 (5.3%)	3 (15.0%)	1 (4.0%)	
2	2 (3.1%)	0 (0.0%)	1 (5.0%)	1 (4.0%)	
Event type (first event)					0.10
Sudden cardiac arrest	1 (1.6%)	1 (5.3%)	0 (0.0%)	0 (0.0%)	
Sustained ventricular arrhythmias	3 (4.7%)	0 (0.0%)	1 (5.0%)	2 (8.0%)	
Appropriate ICD shock	3 (4.7%)	0 (0.0%)	3 (15.0%)	0 (0.0%)	

* Post hoc analysis of follow-up time: *p* values were adjusted by Bonferroni correction for multiple tests with the asymptotic significant level of 0.05. (^a^ Paroxysmal–Persistent, ^b^ Paroxysmal–Permanent, ^c^ Persistent–Permanent).

## Data Availability

The datasets presented in this article are not readily available because the data are part on multiple ongoing studies. Requests to access the datasets should be directed D.S.
